# Contents of the inguinal canal: identification by different imaging methods

**DOI:** 10.1590/0100-3984.2020.0006

**Published:** 2021

**Authors:** Nelson Marcio Gomes Caserta, Thiago José Penachim, Ewandro Braz Contardi, Rayssa Clara Fonseca Barbosa, Thaisa Lazari Gomes, Daniel Lahan Martins

**Affiliations:** 1 Universidade Estadual de Campinas (Unicamp), Campinas, SP, Brazil.; 2 Centro Radiológico Campinas, Campinas, SP, Brazil.

**Keywords:** Urinary bladder, Inguinal canal, Inguinal hernia, Testicular neoplasms, Metastasis, Ovary, Bexiga urinária, Canal inguinal, Hérnia inguinal, Neoplasias testiculares, Metástase, Ovário

## Abstract

Although the correct diagnosis of inguinal hernias can often be made by clinical examination, there are several situations in which imaging methods represent the best option for evaluating such hernias, their content, and the possible complications. In addition, bulging of the inguinal region is not always indicative of a hernia, because other lesions, including tumors, cysts, and hematomas, also affect the region. The objective of this pictorial essay is to demonstrate what can be identified within inguinal hernias. Differentiating the types of herniated structures is of absolute importance for planning the appropriate treatment.

## Abstract (pt)

Embora o diagnóstico correto possa ser muitas vezes realizado pelo exame clínico, há várias situações em que os métodos de imagem representam a melhor opção para avaliar hérnias inguinais, seu conteúdo e eventuais complicações. Além disso, os abaulamentos da região inguinal nem sempre são hérnias, pois outras lesões como tumores, cistos e hematomas também acometem este sítio. O objetivo deste ensaio é demonstrar alguns diferentes conteúdos que podem ser diagnosticados no interior de hérnias inguinais. A diferenciação dos tipos de estruturas herniadas é de suma importância no planejamento do tratamento.

## INTRODUCTION

The inguinal canal is a complex diagonal passage in the lower abdomen that is delimited by the aponeuroses of three muscles: the external oblique, the internal oblique, and the transversus abdominis. In males, the inguinal canal is a passage for the spermatic cord (from the scrotum to the pelvis); in females, it contains the round ligament of the uterus and the ilioinguinal nerve^(1)^.

Inguinal hernias develop when there is failure of neonatal obliteration of the processus vaginalis or, in adults, when elastic and collagen fibers become weakened. Such hernias can be classified as direct or indirect, depending on their position (medial or lateral) in relation to the lower epigastric artery. 

Normal anatomical structures, such as the small intestine, colon, bladder, appendix, ovaries, and testicles, can protrude into the inguinal canal and be subject to various complications and neoplastic or non-neoplastic lesions.

Although inguinal hernias are a common finding, other, less common, conditions can be found in the inguinal canal. Acute processes such as abscesses and hematomas can extend into the canal. Rare complications of disorders such as metastases, ovarian cysts, and bladder carcinomas can be difficult to diagnose when they occur in the inguinal canal^(1)^. 

The objective of this pictorial essay is to show the various structures and lesions that can be localized in the inguinal canal and the imaging methods that can be used in order to establish the diagnosis and inform decisions regarding the appropriate treatment.

## CYST OF THE CANAL OF NUCK

**Figure 1:**
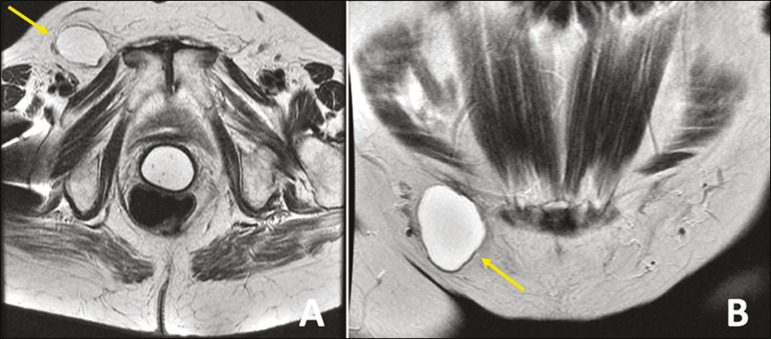
Axial and coronal T2-weighted MRI sequences (**A** and **B**, respectively) showing a lesion with a cystic aspect in the right inguinal canal, with thin walls, no septa, and no solid components, consistent with a cyst of the canal of Nuck. Image courtesy of Dr. Fernando Mansano.

The canal of Nuck is a dilatation of the peritoneum that follows the round ligament and extends from the inguinal canal to the vulva. It is an embryological remnant of the processus vaginalis that remains patent. The partial obliteration of the proximal portion with a patent distal portion leads to the formation of a cyst of the canal of Nuck^(2)^, also known as female hydrocele (Figure 1).

## INCARCERATED INGUINAL HERNIA

**Figure 2:**
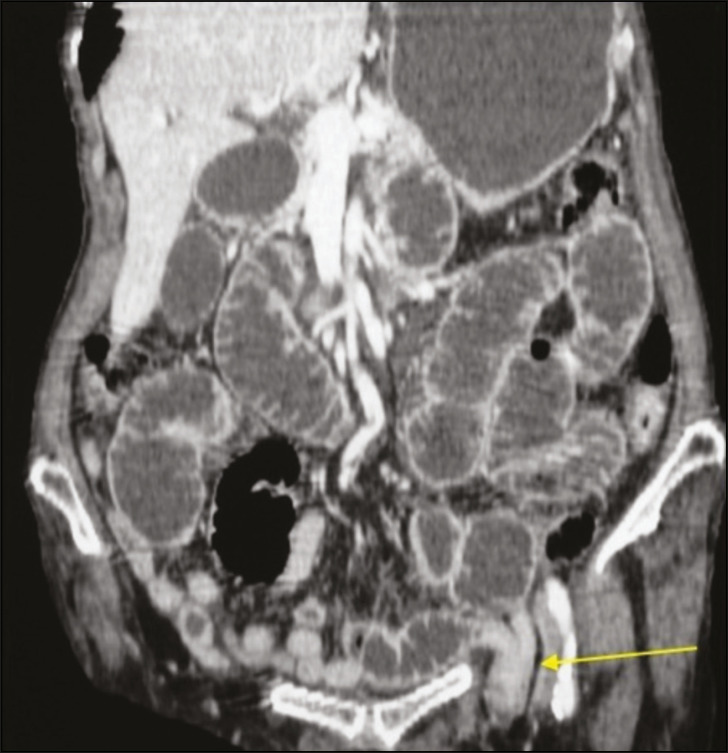
Coronal CT in the portal phase showing an incarcerated left inguinal hernia containing a jejunal loop, complicated by intestinal obstruction. Note the distention of the proximal bowel loops.

Inguinal hernias should be corrected by elective surgery to prevent complications. Nevertheless, incarcerated and strangulated hernias represent a common cause of acute abdomen (Figure 2). 

## INGUINAL HERNIA WITH ASCITES IN PATIENTS WITH CIRRHOSIS

**Figure 3:**
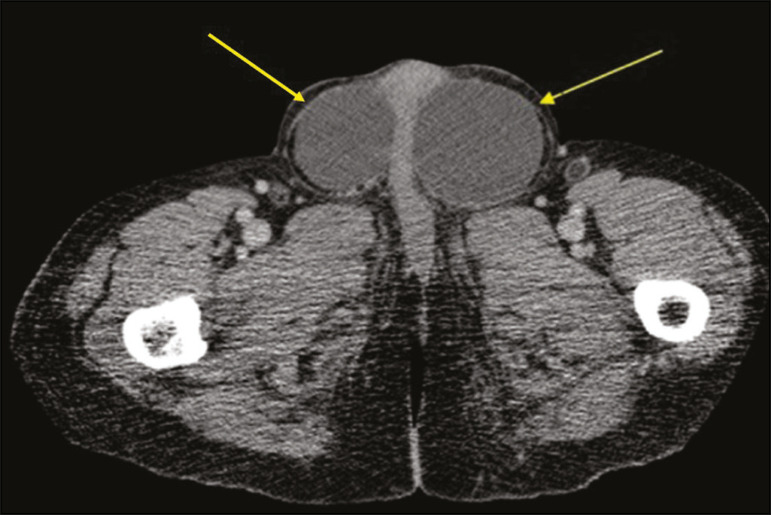
Unenhanced axial CT in a patient with cirrhosis, showing ascites and large inguinoscrotal hernias containing ascitic fluid.

The incidence of hernias in the abdominal wall is high in patients with cirrhosis, especially in those with ascites. An increase in intra-abdominal pressure results in the formation of massive inguinoscrotal hernias (Figure 3). 

## INGUINAL HERNIA CONTAINING THE SIGMOID COLON

**Figure 4:**
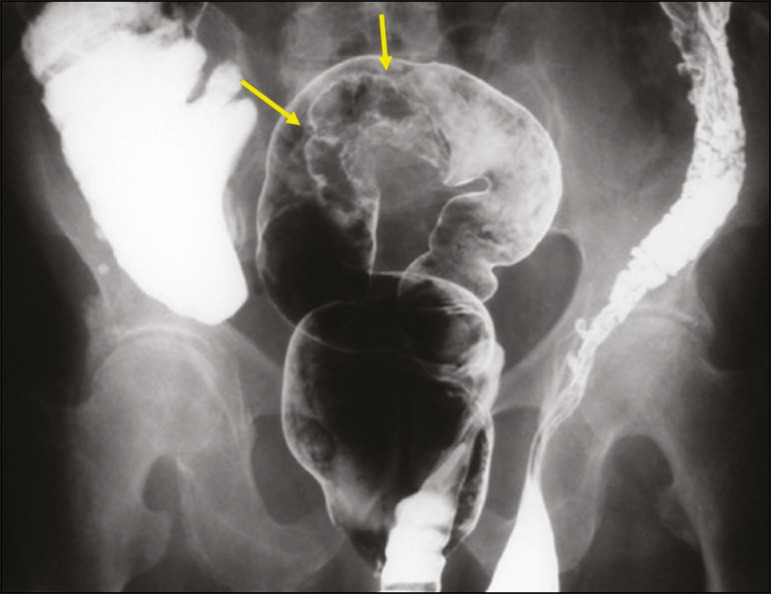
Double contrast-enhanced barium enema showing the sigmoid colon within a left inguinal hernia with poorly filled irregular contours (arrows) that was found to be a carcinoma.

Occasionally, the sigmoid colon becomes trapped within an inguinal hernia. Radiologists should be aware of any accompanying complications such as diverticular disease and primary tumors (Figure 4).

## PERITONEAL CARCINOMATOSIS IN THE HERNIA SAC

**Figure 5:**
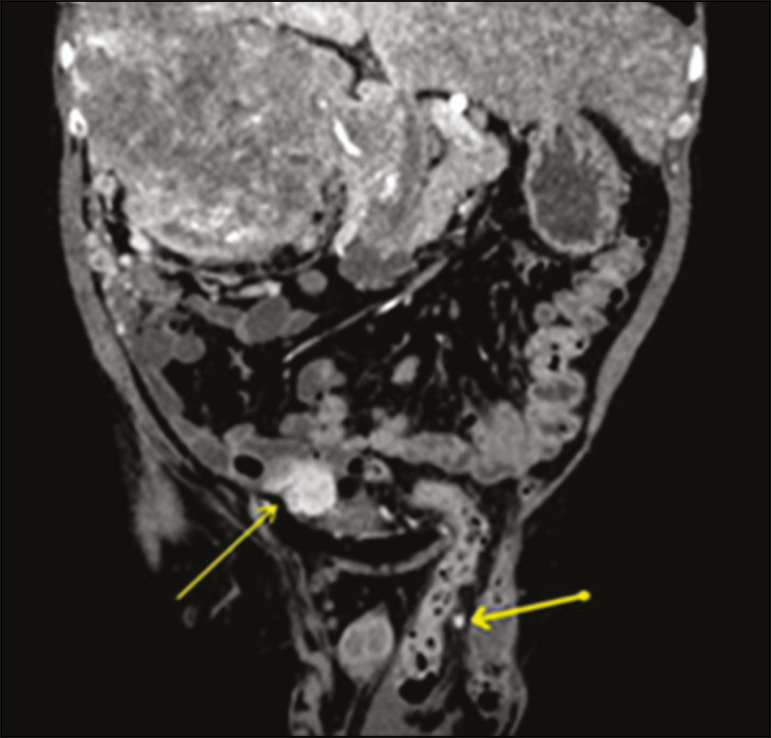
Coronal CT in the arterial phase showing a massive renal cell carcinoma that was hypervascularized on the right, together with nodules typical of peritoneal carcinomatosis, one of them in the left hernia sac (arrows).

The inguinal region can harbor primary or metastatic malignant tumors (Figure 5). Metastases to inguinal lymph nodes most often originate from tumors in the lower portion of the vagina, distal rectum, vulva, anus, or lower limbs. Peritoneal carcinomatosis is an uncommon finding that changes the staging of tumors and should be screened for, even within hernias. 

## EXTENSION OF TESTICULAR LYMPHOMA

**Figure 6:**
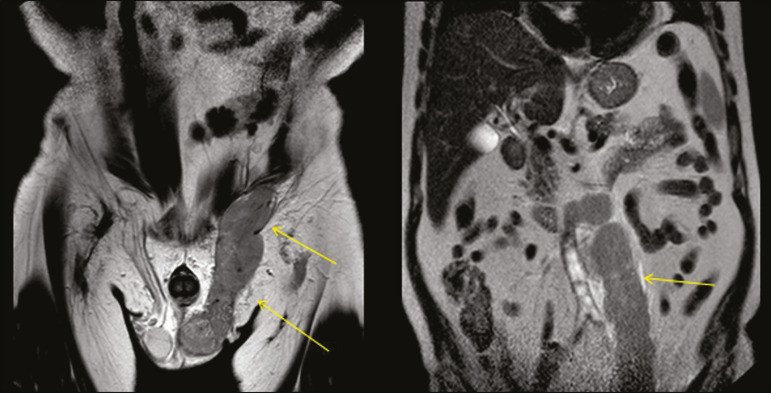
Coronal T2-weighted MRI sequence showing primary testicular lymphoma extending through the inguinal canal and the left gonadal vein (arrows).

Primary testicular lymphoma is an uncommon diagnosis, and its contiguous extension along the spermatic cord, inguinal canal, and gonadal vein is an even rarer manifestation. Figure 6 depicts a case of extension of primary testicular lymphoma.

## AMYAND’S HERNIA

**Figure 7:**
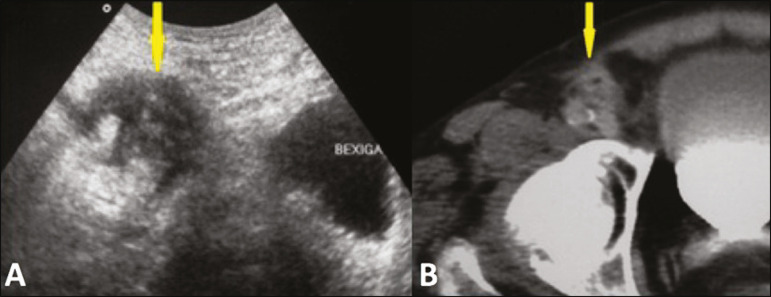
**A**: Axial ultrasound showing an ill-defined, hypoechoic, heterogeneous area in the inguinal canal. **B**: Axial CT of the same patient, showing a thickened appendix in the inguinal canal.

The presence of a cecal appendix, inflamed or not, inside a hernia sac is known as Amyand’s hernia (Figure 7). Acute appendicitis within an inguinal hernia occurs in only 0.1% of all cases, and there is no evidence that Amyand’s hernia increases the risk of appendicitis^(4)^. 

## OVARY IN THE INGUINAL CANAL

**Figure 8:**
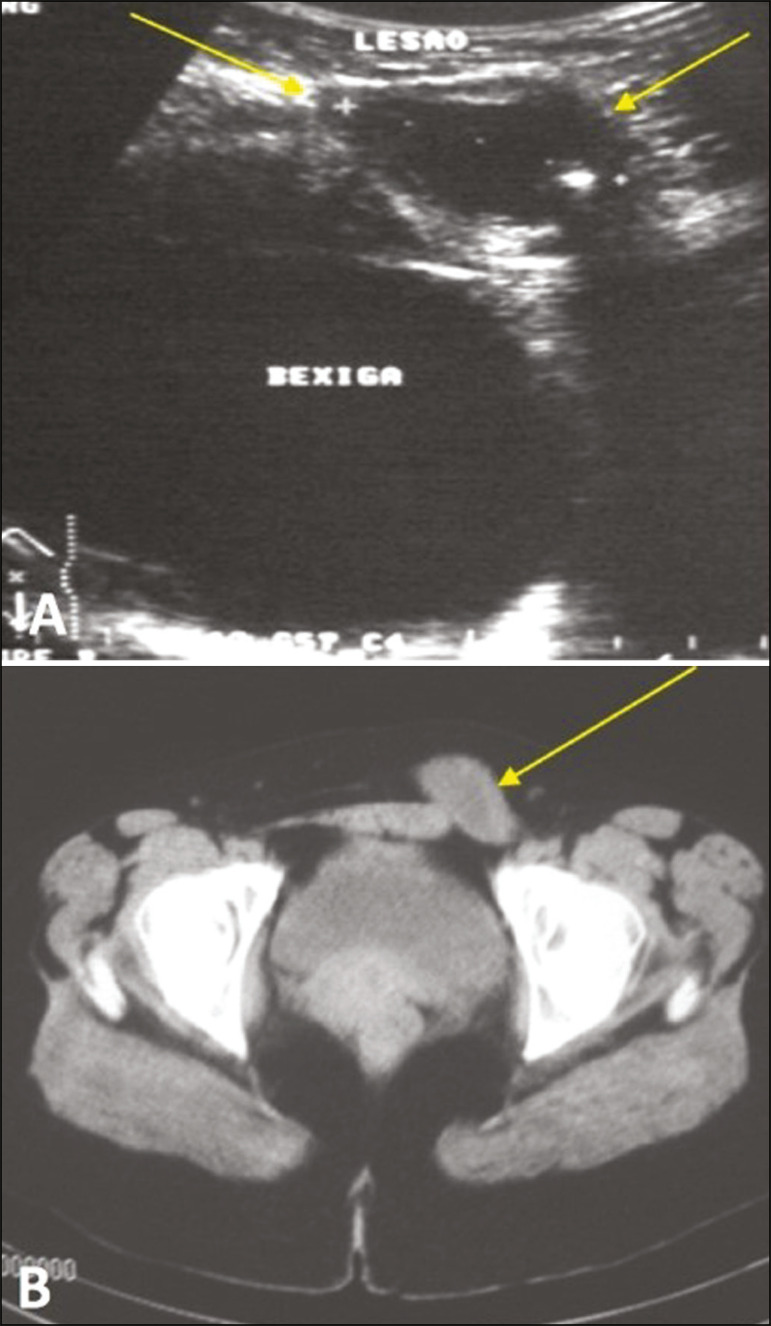
A 28 year-old female with a painful mass, which proved to be an ovarian cyst within an inguinal hernia, in the left inguinal region. **A**: Ultrasound showing an ovarian cyst in the inguinal canal. **B**: Axial CT in the portal phase showing that cyst (arrow). Image courtesy of Dr. Lutero Marques de Oliveira.

An ovary can occasionally be within an inguinal hernia in a neonate and can be identified on physical examinations as a palpable, painless, irreducible mass or, in rarer cases, as a painful bulge that is edematous due to incarceration. In pre-menopausal females, herniation of an ovary, as depicted in Figure 8, is extremely rare^(5)^.

## BLADDER HERNIATION

**Figure 9:**
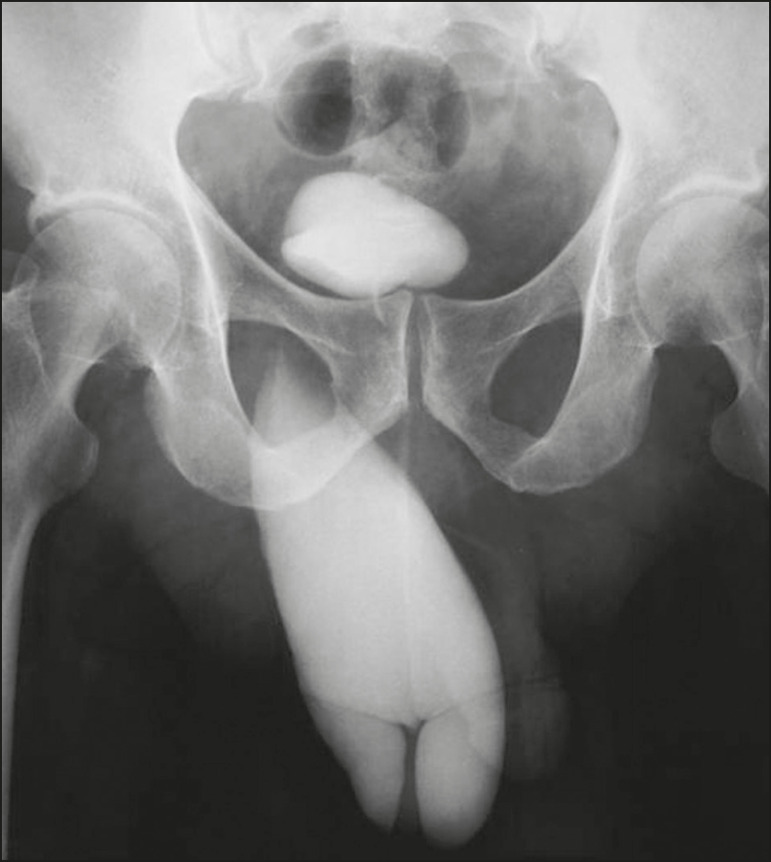
Cystourethrography showing a right inguinal hernia containing part of the bladder visible only in the post-micturition phase. Image courtesy of Dr. Paulo Wiermann.

**Figure 10:**
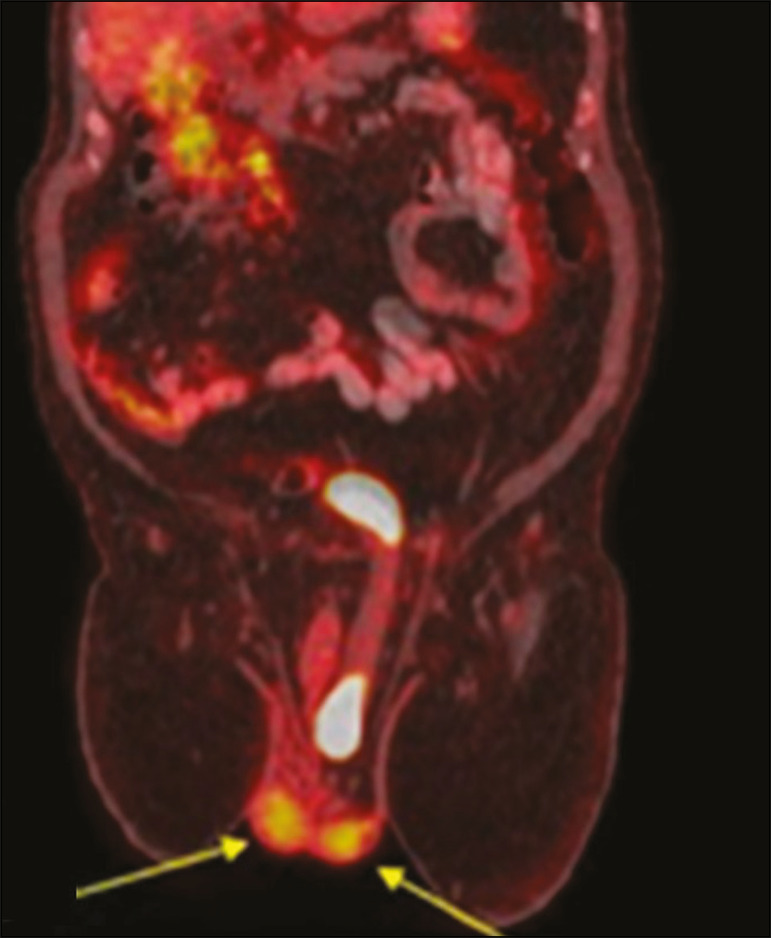
^18^F-FDG-PET/CT fusion image showing the normal testicles (arrows) and a left inguinal hernia containing the bladder, in which there was physiological accumulation of the radiopharmaceutical.

**Figure 11:**
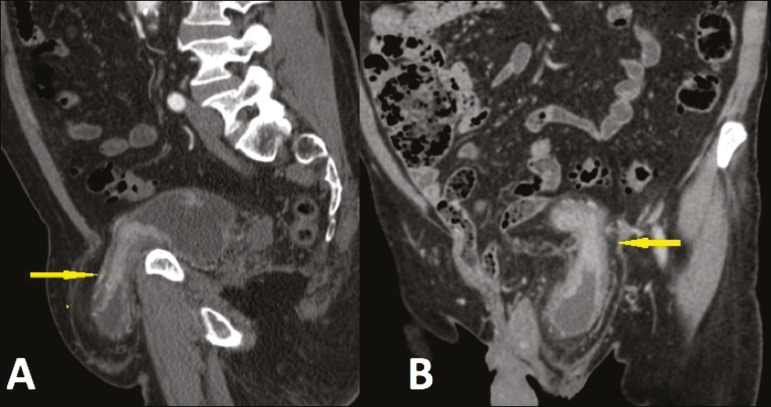
Sagittal and coronal CT scans (**A** and **B**, respectively) in the arterial phase, showing left inguinal herniation of the bladder. Note the area of irregular wall thickening and hypervascularization that proved to be a urothelial carcinoma.

**Figure 12:**
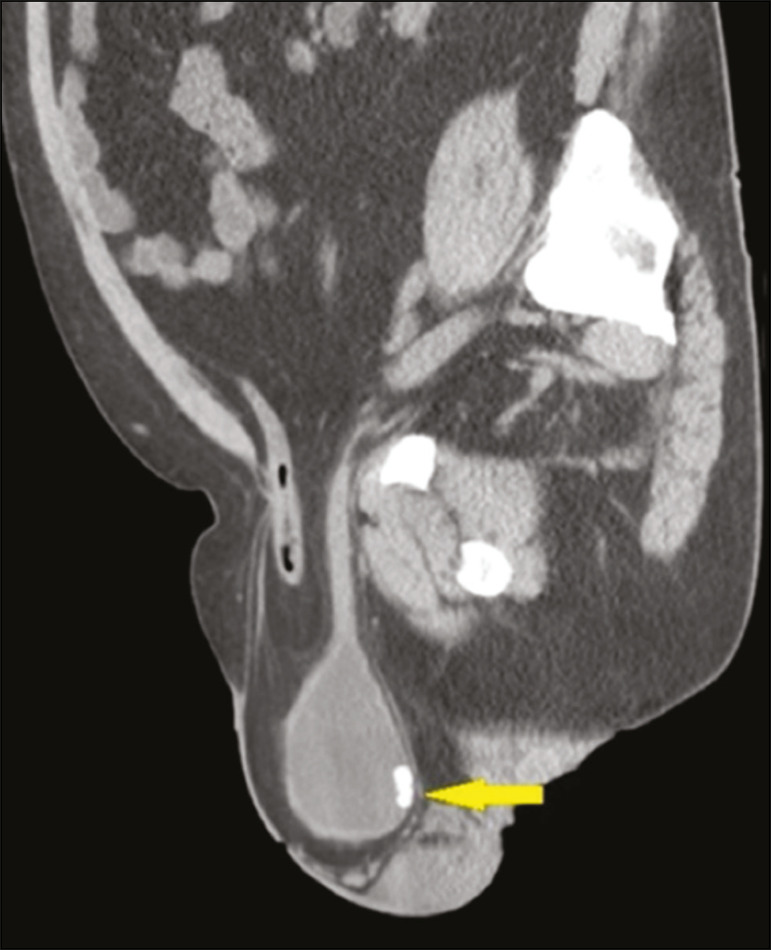
Unenhanced sagittal CT showing a hernia containing an intestinal loop and part of the bladder, together with a calculus (arrow) in the herniated portion.

Approximately 1-3% of all inguinal hernias contain the bladder, part of it, or a diverticulum^(6)^. Some bladder hernias are visible only on post-micturition images (Figure 9). During an ^18^F-fluorodeoxyglucose positron emission tomography/CT (^18^F-FDG-PET/CT) examination, the radiopharmaceutical can accumulate in the portions of the bladder that are within the hernia sac (Figure 10). Tumors and calculi can be seen within a herniated bladder (Figures 11 and 12). 

## HEMATOMA

**Figure 13:**
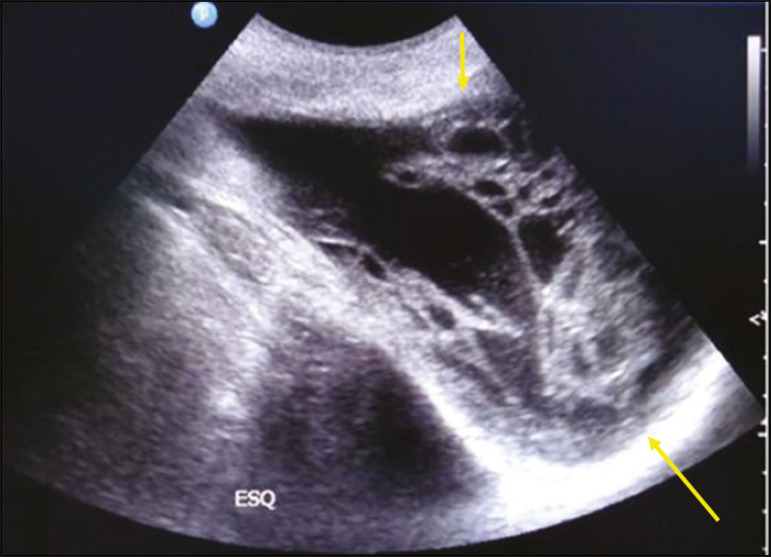
Inguinoscrotal hematoma as a complication of inguinal herniorrhaphy. Ultrasound showing a multilocular formation with heterogeneous, hyperechoic components (arrows).

Hematomas in the inguinal region can occur as a result of anticoagulation, surgery, catheterization, or neoplasia (Figure 13). 

## ABSCESS

**Figure 14:**
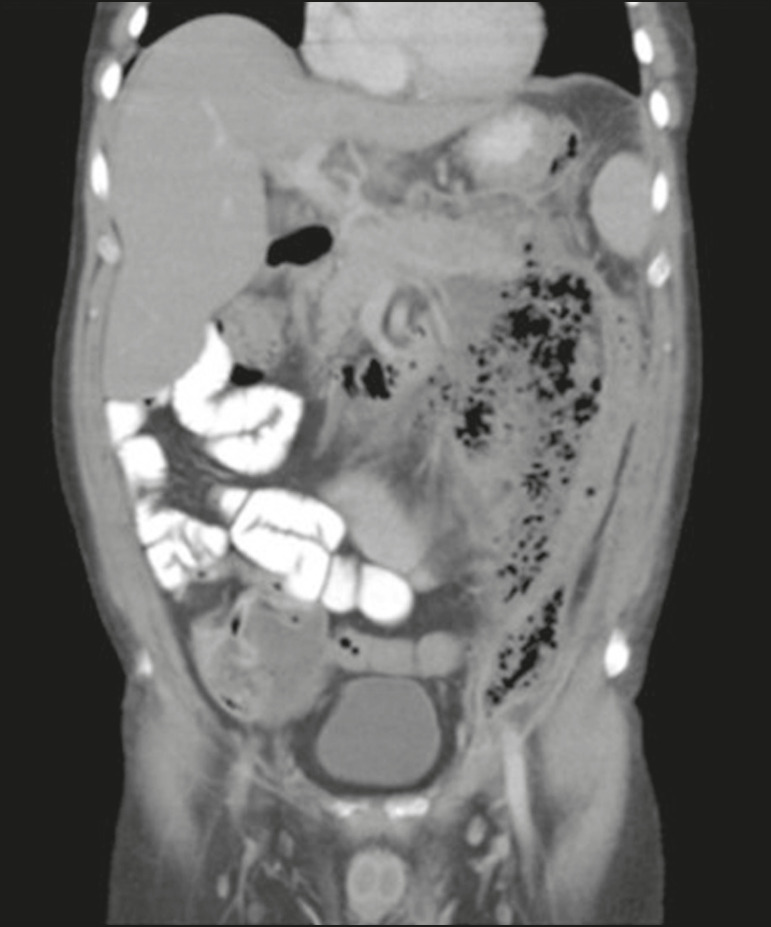
A 45 year-old male with hypertriglyceridemia and alcohol use disorder. Oral contrast-enhanced coronal CT, in the portal phase, showing acute pancreatitis complicated by an abscess invading the left inguinal canal.

**Figure 15:**
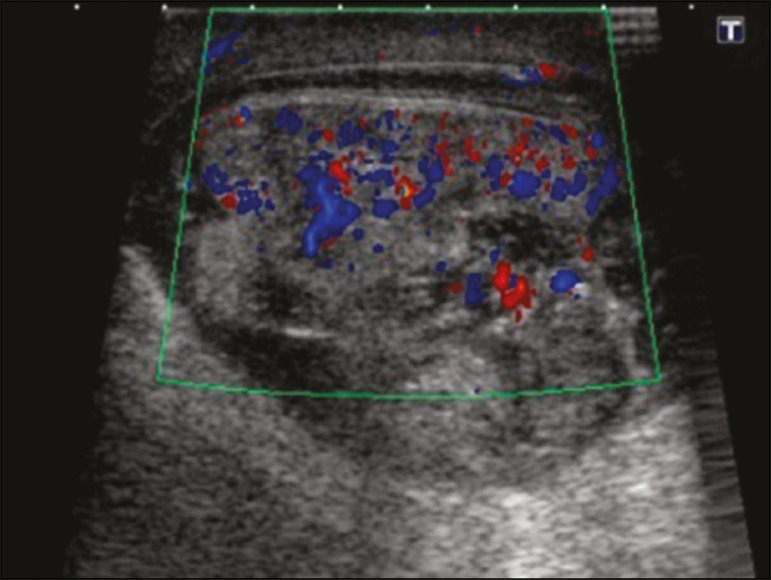
Ultrasound of the same patient depicted in Figure 14, showing a contiguous, heterogeneous fluid collection in the left scrotum, together with fluid collections in the pancreas and groin.

Many conditions, such as incarcerated hernia, Amyand’s hernia, and diverticulitis, can lead to the formation of an abscess in the inguinal canal. Pancreatitis accompanied by a fluid collection, pseudocyst, or abscess extending to the inguinoscrotal region, as shown in Figures 14 and 15, is rare^(7)^.

## CONCLUSION

Knowledge of the various presentation forms of inguinal hernias, contents of such hernias, and potential complications is critical for their correct diagnosis and appropriate treatment. Diagnostic imaging methods play an essential role in this process, and radiologists should be familiar with the relevant findings.
